# Maize’s origin to be revisited

**DOI:** 10.1080/15592324.2024.2332017

**Published:** 2024-03-21

**Authors:** Khaled Moustafa

**Affiliations:** The Arabic Preprint Server (ArabiXiv)

Maize (Zea mays) is one of the world’s most important cereal crops, alongside wheat, barley, and rice. They serve as a staple food for both human and animal populations. Extensive studies have focused on enhancing maize yields and improving its tolerance to environmental biotic and abiotic stresses. For example, the application of brassinosteroids promotes maize growth, yield, and resilience to stress factors.^1^ However, the origin of maize has long been a topic of controversial debate. Many studies suggest that maize is derived from a wild grass known as teosinte, indigenous to Guatemala and Mexico.^2–6^ The maize has then spread from these regions to the Americas, Europe, and other parts of the world following Christopher Columbus’s discovery of America in 1492 CE. Nonetheless, intriguing clues from pre-Columbian Arabic texts cast a shadow of doubt on this narrative. One such book is a botanical and pharmacological work titled “*Compendium on Simple Medicaments and Foods*” written by Ibn Al-Baytar, a renowned Arab-Spanish botanist born in Spain in 1190 CE and passed away in Damascus in 1248 CE.^7^ He wrote his book between 1240 and 1248, namely more than 244 years before the discovery of America in 1492. A free copy of this book is available in two volumes at the Library of the Spanish Agency for International Development Cooperation (AECID).^1^ In this book, recognized as one of the best books in *Materia Medica* in its time,^8^ Ibn Al-Baytar described up to 1400 plant species with their nutritional and medical uses. He described maize with the same name in Arabic, “*thura*,” a term still used to describe the maize as we know it today. His description portrays maize as “*a species of cereal growing with a stem much thicker than that of wheat and barley, and its leaves thicker and wider than theirs*”. This observation suggests that maize may have been known in regions outside of the Americas.

Another significant Arabic text that references maize is a renowned Arabic encyclopedic dictionary titled “Lisan Al-Arab” (The Tongue of Arabs), authored by Ibn Manzur in 1291 CE, nearly two centuries before the discovery of America. In this influential work, Ibn Manzur defines maize as: “*a well-known grain species characterized by leaves similar to those of reed or cane. Its fruit comprises seeds enclosed in red sleeves, which are edible and used in bread-making*”. An additional description of maize can also be found in this book under the entry “*Sakara*” (meaning “get drunk”) to defines a beverage as “*an alcoholic drink made from maize*”. Other ancient Arabic texts such as “Al Kamous Al-Muhit”, another renowned Arabic dictionary predating the discovery of America, also mentions maize as a “well-known grain”, with the verb “*Tahfala*” in Arabic meaning: “eating the bread of maize constantly”.

These descriptions of maize in ancient Arabic texts before the discovery of America, coupled with its consistent name “*thura”* over centuries, implies a preexisting familiarity with the crop in the region. However, it’s worth noting that the term “maize” (thura in Arabic) is used interchangeably to describe both the sorghum, referred to as “white maize” and the corn, known as “yellow maize”. This linguistic interchangeability could potentially introduce confusion that needs further investigation to be elucidated when discussing maize’s historical presence in the region. In Arab countries, however, the term “yellow maize” is commonly referred to as “Shami maize”, where “Shami” denotes the geographical region comprising Jordan, Lebanon, Palestine, and Syria, suggesting maize’s historical presence in this region. Otherwise, if maize had been a newly introduced species to the region, it would likely have retained its original name, as seen with other introduced plant species like the tomato (pronounced in Arabic as “tomat” or “tamatem”) and potato (pronounced “patates” or “patata”). Maize may not have been extensively cultivated in these areas historically but grew in the wild and served various purposes. It may have been utilized primarily as a forage crop, featuring smaller seeds compared to modern varieties, and occasionally incorporated into bread-making, as referenced in Ibn Manzur’s works.

In short, while many studies trace the origins of maize back to Central America, it’s essential to broaden research on its origin to encompass other regions and languages. The story of maize’s origins may be more complex than previously thought. The historical texts mentioned earlier provide glimpses of maize’s presence beyond Central America. Extensive historical and biological research is needed to validate this hypothesis and emphasize the importance of exploring diverse sources of information for deeper insights into maize’s history. It’s plausible that maize domestication occurred in multiple regions with similar climate conditions suitable for its growth and cultivation. The predominant focus on English-language studies and Mexico as maize’s sole origin may overlook valuable information in other languages and regions. Therefore, comprehensive research efforts are necessary to uncover the global domestication of maize and establish scientific and historical truths. Whether maize has originated in a single location or multiple regions, it is the scientific truth that should matter most. A deep understanding of maize’s domestication enriches our scientific knowledge and deepens our appreciation of its cultural and historical significance. The essence of science lies in fostering constructive debates and revealing truths, regardless of external influences. Through studies, debates, and counterarguments, scientific truths can be reached out. Scientific knowledge is constructed upon factual evidence and proofs. The observations highlighted here may catalyze additional research to validate or challenge the origins of maize.

## Note

1.
https://bibliotecadigital.aecid.es/bibliodig/es/consulta/registro.cmd?id=1438
